# On the use of wearable sensors as mobility biomarkers in the marketing authorization of new drugs: A regulatory perspective

**DOI:** 10.3389/fmed.2022.996903

**Published:** 2022-09-21

**Authors:** Marco Viceconti, Maria Tome, Wilhelmus Dartee, Igor Knezevic, Sabina Hernandez Penna, Claudia Mazzà, Brian Caulfield, Judith Garcia-Aymerich, Clemens Becker, Walter Maetzler, Thierry Troosters, Basil Sharrack, Giorgio Davico, Solange Corriol-Rohou, Lynn Rochester, the Mobilise-D Consortium

**Affiliations:** ^1^Department of Industrial Engineering, Alma Mater Studiorum - University of Bologna, Bologna, Italy; ^2^Medical Technology Lab, IRCCS Istituto Ortopedico Rizzoli, Bologna, Italy; ^3^European Medicine Agency, Amsterdam, Netherlands; ^4^Regulatory Affairs, Novartis Pharma AG, Basel, Switzerland; ^5^R&D, Regulatory Affairs, Bayer AG, Wuppertal, Germany; ^6^Department of Mechanical Engineering and Insigneo Institute for in Silico Medicine, University of Sheffield, Sheffield, United Kingdom; ^7^Insight Centre for Data Analytics, School of Public Health Physiotherapy and Sport Science, University College Dublin, Dublin, Ireland; ^8^ISGlobal, Barcelona, Spain; ^9^Departament de Ciéncies Experimentals i de la Salut, Universitat Pompeu Fabra, Barcelona, Spain; ^10^CIBER Epidemiología y Salud Pública (CIBERESP), Madrid, Spain; ^11^Department of Geriatric Medicine, Robert Bosch Gesellschaft für Medizinische Forschung mbH, Stuttgart, Germany; ^12^Department of Neurology, University-Hospital Schleswig-Holstein, Kiel University, Kiel, Germany; ^13^Department of Rehabilitation Sciences, KU Leuven, Leuven and Pulmonary Rehabilitation, University Hospital Leuven, Leuven, Belgium; ^14^Pulmonary Rehabilitation, University Hospital Leuven, Leuven, Belgium; ^15^Department of Neuroscience and Sheffield NIHR Translational Neuroscience BRC, Sheffield Teaching Hospitals NHS Foundation Trust and University of Sheffield, Sheffield, United Kingdom; ^16^AstraZeneca R&D, Global Regulatory Excellence, Paris, France; ^17^Translational and Clinical Research Institute, Faculty of Medical Sciences, Newcastle University, Newcastle upon Tyne, United Kingdom

**Keywords:** digital mobility outcomes, regulatory qualification, mobility biomarkers, wearable sensors, mobility disability

## Abstract

The loss of mobility is a common trait in multiple health conditions (e.g., Parkinson's disease) and is associated with reduced quality of life. In this context, being able to monitor mobility in the real world, is important. Until recently, the technology was not mature enough for this; but today, miniaturized sensors and novel algorithms promise to monitor mobility accurately and continuously in the real world, also in pathological populations. However, before any such methodology can be employed to support the development and testing of new drugs in clinical trials, they need to be qualified by the competent regulatory agencies (e.g., European Medicines Agency). Nonetheless, to date, only very narrow scoped requests for regulatory qualification were successful. In this work, the Mobilise-D Consortium shares its positive experience with the European regulator, summarizing the two requests for Qualification Advice for the Mobilise-D methodologies submitted in October 2019 and June 2020, as well as the feedback received, which resulted in two Letters of Support publicly available for consultation on the website of the European Medicines Agency. Leveraging on this experience, we hereby propose a refined qualification strategy for the use of digital mobility outcome (DMO) measures as monitoring biomarkers for mobility in drug trials.

## Introduction

The loss of *mobility* (physical mobility, the ability to move freely and easily without a vehicle, e.g., the ability to carry out ambulatory activities) is a common consequence of multiple health conditions. The consequences of mobility loss are significant and wide reaching, leading to loss of independence and quality of life and being associated with future adverse events such as falls.

Loss of mobility can take different forms for different diseases, which highlights the need to understand mobility not as a single variable but as a combination (pattern) of mobility constructs. For example, multiple sclerosis (MS) is a chronic demyelinating disease of the central nervous system. MS affects different clinical domains including ambulation; gait is one of the most important and valued functions for patients with MS and its dysfunction is one of the main contributors to poor quality of life in such patients (1). Chronic obstructive pulmonary disease (COPD) refers to a group of airway diseases that cause airflow obstruction and breathing-related problems. In addition to the respiratory problems, patients also suffer from “systemic” consequences of the disease, which include for example, muscle weakness. Physical activity and mobility limitations are a cardinal feature of COPD (2). Proximal Femoral Fracture (PFF) is the most debilitating type of fragility bone fracture. More than 800,000 PFF are reported every year in the EU-28 region (3). Over 40% of PFF patients do not regain pre-fracture mobility levels. Parkinson's Disease (PD) is a progressive central nervous system disorder that affects movement; early symptoms of PD include rigidity, tremor, slowness of movement, and difficulty in walking (4).

Thus, the Mobilise-D consortium selected MS, COPD, PFF and PD which despite different etiopathogenesis and disease presentation patterns, are all known to significantly impact patients' mobility. PFF produces a sudden and total loss of mobility in usually older patients (i.e., over 80), who then recover their locomotor function in variable degrees and at different speed. MS, which is usually diagnosed in people in their 20–40 s, is characterized by flares, and progresses quite heterogeneously between patients. PD is usually diagnosed in patients over 60 years old and tends to have a relatively steady progression. COPD, which is commonly misdiagnosed, may not be diagnosed until the disease is advanced, on average in people around 40 years old, and is known to have little impact on mobility in its early stages. Thus, these four diseases are a good sample of the wide range of modalities with which the loss of mobility manifests across diseases.

As mobility can be a fairly vague concept, we propose the following definitions:

- **Mobility**—physical mobility, the ability to move freely and easily without a vehicle. Mobility can be evaluated in terms of capacity, patient's perception, and performance.
- **Mobility capacity**—intensity with which the patient can perform an assigned motor task, as assessed within a clinical assessment.
- **Mobility perception**—mobility as it is perceived by the patients themselves in their daily life; mainly assessed with questionnaires and diaries.
- **Mobility performance**—mobility measured in the real world and over a sufficiently long period of time, for example with wearable digital devices.
- **Mobility disability**—loss of mobility performance. Mobility Performance is a monitoring biomarker of the construct Mobility Disability. Mobility Capacity and Mobility Perception can be used as surrogate biomarkers of the construct Mobility Disability.
- **Mobility-related activities of daily living**—ADL are those activities that allow an individual to live independently in a community. Of the seven ADL normally considered six are related to mobility: Bathing and Grooming, Dressing and Undressing, Meal Preparation and Feeding, Functional Transfers, Safe Restroom Use and Maintaining Continence, and Ambulation.
- **Digital mobility outcomes**—DMOs are digitally measured outcomes used to assess an individual's mobility, e.g., in terms of gait spatiotemporal features, walking bout characteristics, and physical activity (duration, quality, and intensity of mobility).

From the patients' perspective, mobility disability is the inability to perform activities of daily life, especially those repeated on a regular basis; ideally, we should monitor mobility in the real world, and over a sufficiently long period of time to capture all repeating patterns in the patient's lifestyle, so to notice such changes. However, to date there are no tools qualified by regulatory authorities to monitor mobility in the real world for sufficiently long time (e.g., a week).

Inertial Measurement Units (IMU) that integrate accelerometers, gyroscopes and magnetometers are in principle capable of measuring the position, velocity, and acceleration of the center of mass of a human body. IMUs became miniaturized enough to be wearable a while ago; however, the signal processing algorithms that quantify mobility outcomes from raw signals generated by the sensors are unreliable for subjects walking slowly, or with unusual gait patterns because of their health condition. The IMI-funded Mobilise-D consortium (5)^1^ is currently validating a new generation of algorithms that offer the potential for accurate and reliable measurement of mobility outcomes even in patients with slow, pathological gait and general loss of physical capacity; thus, the consortium approached the European Medicines Agency (EMA) to identify the process required for the regulatory qualification that would allow the use of mobility in the real world to generate evidence for inclusion in the marketing authorization of new medicines. But the qualification of such complex methodology as a methodology/tool for clinical drug development is challenging and poses relevant regulatory and scientific problems. Various attempts were made so far with EMA and the US Food and Drug Administration (FDA), which were unsuccessful, or extremely limited in scope (6). Because of the complexity of the topic, the Mobilise-D consortium agreed with the EU regulators to adopt a staggered strategy. Therefore, the Mobilise-D consortium made two consecutive requests for qualification advice to EMA. The first was submitted on 04 October 2019 and requested qualification advice for the use of DMOs as monitoring biomarkers of the loss of mobility in patients affected by PD. The submission and the resulting advice are summarized in a Letter of Support published by EMA on its website in April 2020^2^, and in greater detail in a subsequent publication (6). The most important result of this first submission was that the proposed design of the technical validation (7), which is disease-independent, device-agnostic, and based on separating the validation of a device from the validation of the analysis of the data collected with the device, was found acceptable by EMA. Importantly, the technical validation activities are now near to completion.

The second request for qualification advice which was submitted in June 2020, was also followed up with a Letter of Support summarizing our second submission in May 2021^3^. The aim of this paper is to provide more details on this second submission, the advice provided by EMA, and the regulatory strategy that is emerging from the collective advice.

## Methods

### Mobilise-D submission

The rationale for seeking regulatory advice was related to a new methodology developed by the Mobilise-D consortium to quantify mobility performance over 7 consecutive days and in a real-world setting, using wearable sensors. The intention is to use the DMOs provided by this new methodology:

- As disease-specific monitoring biomarkers to account for mobility performance in assessing the efficacy of new treatments for patients affected by COPD, MS, and PFF, in addition to PD patients as discussed in the initial Qualification Advice (EMA/CHMP/SAWP/128601/2020). These new monitoring biomarkers will complement those already in use, that account for a patient's perception of mobility (e.g., the PPACs—PROactive Physical Activity in COPD—developed and qualified in 2018 by the IMI PROactive in COPD consortium^4^) or mobility capacity.
- As disease-independent monitoring biomarkers to account for mobility performance in assessing the efficacy of new treatments for patients affected by PFF, COPD, MS, and PD, that all impact mobility performance.
- As surrogate endpoints, to replace currently used and accepted (hard) clinical endpoints that are more difficult, expensive, or time-consuming to use than the DMOs.

The briefing book posed three key questions to EMA:

1) Does the EMA agree that the clinical validation of the use of DMOs as disease-specific monitoring biomarker of mobility performance for each of the three additional diseases under consideration (COPD, MS, and PFF) is fit for purpose?
2) Does the EMA agree that the clinical validation of the use of DMOs as potentially generic and disease-independent monitoring biomarkers of mobility performance for all four diseases under consideration (PD, COPD, MS, and PFF) is fit for purpose?
3) The Applicant plans to perform a 24-month observational study to support the validation of disease-specific DMOs in four disease areas and of disease-independent DMOs to assess the mobility performance of patients. Does the EMA agree with the study design (incl. patient characteristics), sample size, endpoints, and approach to evaluate responsiveness?

For the clinical validation study, the Consortium proposed a 24-month longitudinal observational cohort study to be conducted in 17 sites located in 10 EU countries. The objective of the study is to identify and validate four disease-specific DMOs and a disease-independent DMO that can assess the mobility performance of the patient. The clinical validation study will use two technically validated devices and a suite of technically validated algorithms to quantify the DMOs. The study will enroll 2400 subjects with PD, COPD, MS and PFF, resulting in four different disease cohorts (with 600 participants in each). To have a generalizable clinical population, the 17 consortium partners provide a good geographical representation across Europe: the cohorts maximize variability in mobility difficulties, provide diverse representation of culture, age, gender, and health care services (in- and out-patient; Beveridge and Bismarck model countries). The primary objective is to assess the capacity of DMOs to predict global and disease-specific clinical outcomes using relevant endpoints:

- Global (all cohorts) endpoint: the Late-Life Functional Disability Index (LLFDI).
- Disease-specific endpoints: fall frequency for patients with PD and MS; occurrence/rate of moderate to severe exacerbations for patients with COPD; and admission to care/nursing home following PFF.

In addition to the general eligibility criteria valid across all cohorts that were selected to enhance subjects' compliance and adherence to the protocol and to minimize bias (e.g., use of walking aids), disease-specific inclusion and exclusion criteria have been defined. This will help focusing on those subgroups of patients who would benefit most from the outcomes of the project, while maximizing inclusiveness (and generalisability), and further enables the stratification of subjects in disease severity subgroups (e.g., according to EDSS score for MS, or H&Y for PD), which is key in the proposed validation plan for all disease specific DMOs.

The proposed clinical validation concept was that each DMO should be tested in terms of construct validity, predictive capacity, and ability to detect change in clinically relevant variables. The Consortium proposed to demonstrate construct validity by showing that DMOs correlate with related constructs, they distribute differently across known (sub)groups of patients expected to have a different mobility performance, and do not correlate with constructs that are not correlated with mobility (e.g., visual impairment). Following the same approach proposed for PD in the first qualification advice request, predictive capacity would be demonstrated by showing that DMOs predict widely accepted clinical endpoints for PFF, COPD and MS. Last, it was proposed to demonstrate that DMOs obtained within the Mobilise-D observational study can detect change by testing longitudinal validity against therapy changes and/or changes in health outcomes meaningful to patients and/or clinicians and calculating the minimal clinically important difference (MCID, sometime also referred to as MID) using meaningful changes in constructs relevant to patients and clinicians as anchors (such as changes in SPPB score or change in LLFDI disability sub-score). The Consortium also proposed to try evaluating the responsiveness of DMOs using the observational study (by using changes in therapies required by the evolution of the disease), before resorting to interventional trials. For the purpose of comparing DMOs across diseases, we proposed to use the Late Life Function and Disability Instrument (LLFDI).

## Results

### EMA CHMP advice

The most important elements of the advice are:

- EMA agreed on the distinction between mobility performance, mobility capacity, and mobility perception.
- EMA agreed that the construct mobility performance could provide valuable additional information about mobility disability in the four diseases of interest.
- EMA supported the overall clinical validation concept.
- Since mobility performance has never been evaluated so far in a regulatory submission, there is no gold standard to be used. The measurements may or may not correlate with other known mobility outcomes such as mobility capacity or mobility perception.
- EMA agreed that, if a MCID of disease specific outcomes is justified, the observational multicentre study may be adequate to identify the most appropriate DMO per disease in terms of its ability to detect change.
- EMA considered the observational multicentre clinical study proposed by the Mobilise-D consortium as an important step toward the validation of DMOs, but only exploratory. With respect to the ability to detect change, EMA considered it necessary to evaluate responsiveness also with at least one interventional randomized clinical trial for each disease of interest.

EMA working party experts believed that monitoring mobility performance in real world settings may provide an additional dimension of mobility, in addition to patient's mobility perception and mobility capacity across the four diseases. But the discussion revealed that it remains unclear which of these dimensions of mobility is perceived as most important by patient and health care professionals.

The disease-specific endpoints proposed by the Mobilise-D consortium to validate the predictive capacity of the DMOs (e.g., EDSS, T25-FW and MSWS-12 for MS; MDS-UPDRS III and II for PD; and FEV_1_, 6MWT and exacerbations for COPD), were recognized as standards for the assessment of the disease status and progression. However, considering that many of these endpoints are themselves evaluations of mobility capacity or mobility perception, a certain degree of circularity was involved.

This became evident in the discussion on how to evaluate the ability to detect change of DMOs. EMA agreed that for PFF admission to long-term care could be a valid anchor; the same applied to exacerbations for COPD, although there is no regulatory experience on how these relate to mobility outcomes. EMA suggested that for MS, EDSS could be a more appropriate anchor than fall frequency, as proposed by the Mobilise-D consortium. In general, the experts seemed to favor the use of non-ambiguous events that could be assumed as true values in the validation of the DMOs, rather than trying to validate predictors with other predictors.

With respect to the goal of validating a single DMO as disease-independent monitoring biomarker for mobility performance, EMA agreed that the decline in LLFDI could be used to this purpose, if multiple DMOs perform similarly across diseases. However, while EMA supported the use of LLFDI for older patients, additional evidence would be required in younger patients and across the four diseases of interest, given the validation status of LLFDI (8–11).

Last, EMA agreed that if the clinical validation was successful, DMOs could be positioned as secondary endpoints in the marketing authorization application for a new drug, for example to support relevant labeling claims for inclusion in the product information.

## Discussion

The aim of this paper was to define, from two consecutive rounds of qualification advice received from EMA, a qualification strategy for the use of DMOs as monitoring biomarkers for mobility in drug trials. In the following, a strategy is presented as a list of hypotheses, followed by a description of the approach to be used to produce the evidence required to support each hypothesis, and to open up interactions with the US FDA.

### Constructs' hierarchy

The following hierarchy of constructs is proposed:

1. Mobility
  1.1 Mobility capacity
  1.2 Mobility perception
  1.3 Mobility performance
    1.3.1 Loss of Mobility Performance (Mobility Disability)

### Hypotheses

The qualification of a new methodology/tool for drug development is organized like the testing of a scientific theory, in the Popperian sense. We start with a set of hypotheses to be tested, and then we propose a qualification plan to the regulatory agency where we will attempt the experimental falsification of each of these hypotheses. If the advice of the regulatory agency is supportive of the proposed qualification plan, and if the results of the experimental studies do not falsify any of the hypotheses, a qualification opinion can be requested, which if positive enables, from then on, the use of the new methodology/tool in any marketing authorization request, without any further scrutiny. Thus, it is important to state the hypotheses that the whole qualification process aims to test:

- **Hypothesis #1**—New algorithms are available that provide accurate and reliable quantification of various DMOs to measure mobility performance in real life over a period of seven days using appropriate wearable IMU sensors.
- **Hypothesis #2**— The loss of mobility performance is perceived by both patients and medical professionals as a very important aspect in all four diseases of interest (MS, COPD, PFF, and PD). Thus, every treatment should be evaluated also on the effect it has on mobility performance, both in terms of efficacy (the drug intends to improve mobility performance, or slow down its progression) and safety (the drug does not cause, as unintentional effect, a decrease in mobility performance).
- **Hypothesis #3**—Selected DMOs when used as monitoring biomarkers of mobility performance are superior to other currently used biomarkers for mobility capacity or mobility perception when these are used as surrogate biomarkers for mobility performance.

Hypothesis #1 requires no further explanation.

The need to provide evidence in support of hypothesis #2 has been stated in various documents from EMA and FDA. While all practitioners in our consortium agree that the ability of moving around in daily life is a key determinant of the quality of life of the patient, no regulatory evidence has been produced so far to support that this is indeed the case.

The rationale behind hypothesis #3 is the following. There is confusion on how to appropriately measure mobility. Such confusion arises because until recently mobility performance could not be measured reliably and thus other biomarkers of mobility, such as mobility capacity and the patient's perception of mobility were used as surrogate biomarkers for mobility performance. However, this relation of surrogacy was rarely made explicit. Our hypothesis is that the DMOs measured with the Mobilise-D methodology will be better at monitoring mobility performance than other indicators such as mobility capacity and mobility perception, which are only surrogate biomarkers of mobility performance in the real world.

### Qualification plan

For a defined context of use, the following qualification plan relies on a technical validation to validate hypothesis #1, a qualitative research program to validate hypothesis #2, and a clinical validation to validate hypothesis #3.

#### Context of use

The Mobilise-D DMOs are used for monitoring mobility in the regulatory evaluation of a new drug in diseases, where mobility disability is an important sign of the disease. Mobilise-D DMOs are used to confirm that the new drug causes a progression of mobility disability that is significantly slower than the placebo or comparator. Mobility disability is a secondary efficacy endpoint of the interventional RCT, as measured with the monitoring biomarker Mobilise-D DMO.

#### Technical validation

The testing of hypothesis #1, relevant for all three proposed contexts of use, requires a detailed technical validation. The technical validation plan (7) was approved unconditionally by EMA in the first qualification advice. The technical validation is conducted with patients affected by the four diseases of interest, as well as with healthy volunteers. To separate the error due to the wearable device from that due to the algorithms that extract the DMOs from the raw signals, we defined a set of spot-checks based on the IEEE 2700–2017^5^ standard for metrological characterization for each IMU model in use. We then quantified the error due to the algorithm through a multistage validation procedure that combined in-lab tests and real-world tests. In each test stage we used a metrological approach: the accuracy of a wearable multi-sensor system [INertial module with DIstance Sensors and Pressure insoles, INDIP (12)] was assessed using a fully validated stereophotogrammetric system. The wearable INDIP system was then used to validate the wearable IMUs in real world conditions. More details on the technical validation can be found here (7).

#### Qualitative research

The testing of hypothesis #2, also relevant for all three proposed contexts of use, requires qualitative research to evaluate the *clinical meaningfulness* of outcomes measures of mobility performance (13). Outcome measures must be valid and reliable and assist us in interpreting change in our patients that is clinically meaningful. Clinically meaningful outcomes directly measure how a patient feels, functions, or survives. *Clinical meaningfulness* is the practical importance of a treatment effect—whether it has a real genuine, palpable, noticeable effect on daily life. Clinical meaningfulness generally refers to an outcome measure's ability to provide the clinician and the patient with consequential information.

To ensure that the DMOs represent outcomes that are meaningful to patients and capture meaningful aspects of their health, the consortium is actively working with patients across our cohorts. As part of the technical validation study a series of semi structured interviews have been carried out with patients from all 4 cohorts, addressing issues such as their perceptions on the meaningfulness of mobility in their lives and the use of digital strategies to measure their mobility performance. Data from these interviews are currently being analyzed. In ongoing work multiple methods are being used, including literature reviews, focus groups, semi structured interviews, and surveys to develop a robust Conceptual Framework to support the quantitative clinical validation plan. We also intend to adopt relevant CTTI recommendations^6^, and the approach proposed by Manta et al. (14).

### Clinical validation

The clinical validation plan has not been published yet, but the key elements can be found here (15). The multicentric observational clinical study will involve 600 patients for each of the four disease groups (MS, PD, PFF, COPD) for a total of 2,400 participants recruited in 17 centers across Europe. Each enrolled patient will be followed-up for 24 months, during which 5 visits will take place; after each visit the patient will wear an IMU for seven days. Using the Mobilise-D algorithms, these recordings will be split into a series of walking bouts, and then each bout will be analyzed to calculate a number of different DMOs [see (5) for a list].

Each DMO, and combinations of DMOs, will be correlated to disease-specific mobility-related outcomes in use such as EDSS for MS or MDS-UPDRS II for PD, and to disease-independent scales like LLFDI and SPPB. While we would expect some correlation with those, our assumption is that Mobilise-D DMOs are a better biomarker of mobility performance than other mobility outcomes that express mobility capacity of mobility perception. Thus, to compare the DMOs to these scales, currently used as surrogate biomarkers of mobility performance, we need a clinical outcome that is strongly correlated to the severity of the mobility disability. The idea is to use changes in Mobility-Related Activities of Daily Living (MRADL) as such clinical outcome. This is consistent with the idea that from a patient's perspective, mobility disability is the loss of specific MRADL in their daily life.

Using the data collected in the observational study, we plan to demonstrate for the Mobilise-D DMOs construct validity and predictive capacity. With respect to the ability to detect change we will try to demonstrate longitudinal validity and calculate the MCID for each disease. Following the EMA advice, to evaluate responsiveness it will be necessary to test the DMOs in an interventional double-blind RCT for each disease of interest.

Following the staged approach recommended by EMA, and thanks to the two qualification advices they provided, we now have a clear qualification plan. The next step is to present such qualification plan to the FDA through the CDER Clinical Outcome Assessment Qualification Program^7^.

## Study highlights

### What is the current knowledge on the topic?

While recent technological improvements now enable to monitor mobility continuously and reliably, the regulatory qualification of digital mobility outcome measures to support labeling claims in the market authorization process has so far been limited.

### What question did this study address?

In this study, the Mobilise-D consortium summarizes its successful interaction with the European Medicines Agency that led to two publicly available Letters of Support on the use of Digital Mobility Outcomes as biomarkers of disease status and progression in four clinical indications.

### What does this study add to our knowledge?

By sharing its positive experience and outlining a revised regulatory strategy resulting from the two Qualification Advices received, the Mobilise-D consortium aims to provide a valuable example and a potential starting point (regulatory strategy) for future requests for regulatory qualification.

### How might this change drug discovery, development, and/or therapeutics?

The regulatory strategy hereby outlined may inform future requests for marketing authorization of new drugs based on evidence gathered *via* mobility biomarkers derived from continuous recordings of wearable sensors.

## Data availability statement

The original contributions presented in the study are included in the article/supplementary material, further inquiries can be directed to the corresponding author/s.

## Author contributions

This study is part of a large collaborative initiative, involving several members of the Mobilise-D consortium. MV, MT, WD, IK, SH, CM, BC, JG-A, CB, WM, TT, BS, GD, SC-R, and LR wrote the manuscript and performed the research. MV, WD, IK, SH, and LR designed the research. All authors contributed to the article and approved the submitted version.

## Funding

This work was supported by the Mobilise-D project that has received funding from the Innovative Medicines Initiative 2 Joint Undertaking (JU) under grant agreement No. 820820. This JU receives support from the European Union's Horizon 2020 research and innovation program and the European Federation of Pharmaceutical Industries and Associations (EFPIA). Content in this publication reflects the authors' view and neither IMI nor the European Union, EFPIA, or any Associated Partners are responsible for any use that may be made of the information contained herein. CM was supported by the NIHR Sheffield Biomedical Research Center (BRC).

## Conflict of interest

The authors declare that the research was conducted in the absence of any commercial or financial relationships that could be construed as a potential conflict of interest.

## Publisher's note

All claims expressed in this article are solely those of the authors and do not necessarily represent those of their affiliated organizations, or those of the publisher, the editors and the reviewers. Any product that may be evaluated in this article, or claim that may be made by its manufacturer, is not guaranteed or endorsed by the publisher.

## Author disclaimer

The views expressed are those of the authors and not necessarily those of the NHS, the NIHR or the Department of Health and Social Care (DHSC).

## Footnotes

^1^ https://www.mobilise-d.eu/

^2^ https://www.ema.europa.eu/en/documents/other/letter-support-mobilise-d-digital-mobility-outcomes-monitoring-biomarkers_en.pdf

^3^ https://www.ema.europa.eu/documents/other/letter-support-mobilise-d-digital-mobility-outcomes-monitoring-biomarkers-follow_en.pdf

^4^ https://www.ema.europa.eu/en/documents/regulatory-procedural-guideline/qualification-opinion-proactive-chronic-obstructive-pulmonary-disease-copd_en.pdf

^5^ https://standards.ieee.org/ieee/2700/6770/

^6^ https://ctti-clinicaltrials.org/recommendations-and-resource/

^7^ https://www.fda.gov/drugs/drug-development-tool-ddt-qualification-programs/clinical-outcome-assessment-coa-qualification-program#:~:text=The%20CDER%20Clinical%20Outcome%20Assessment,guiding%20COA%20development%20for%20qualificatio
